# Energy-saving mechanism research on beam-pumping unit driven by hydraulics

**DOI:** 10.1371/journal.pone.0249244

**Published:** 2021-04-01

**Authors:** Hukun Yang, Jianping Wang, Hui Liu

**Affiliations:** 1 School of Mechanical Engineering, Chaohu University, Hefei, Anhui, China; 2 Ironman Academy, Daqing, Heilongjiang, China; 3 School of Mechanical Science and Engineering, Northeast Petroleum University, Daqing, Heilongjiang, China; University of Genova, ITALY

## Abstract

Aiming to solve the problems of long transmission chain, large movement inertia of components and high energy consumption of pumping units, this proposes a new pumping unit with direct balance and hydraulic drive. Through mathematical modeling and simulation analysis to compare the suspension dynamic characteristics and balance characteristics of the hydraulically driven pumping unit with the conventional one. It turns out that the suspension maximum speed drop 21.14%, the maximum acceleration drops 28.88% and the root mean square torque drops 92.9% on the suspension of the hydraulically driven pumping unit. The experimental results proves that the hydraulically driven pumping unit has significant energy saving efficiency, and achieves more than 30.9% of active power saving rate. Theoretical and practical research results show that hydraulically driven pumping unit is reliable and better energy saving, which provides a basis in theory and engineering practice in application.

## Introduction

The conventional beam-pumping unit is durable and reliable. However, due to its four-bar linkage structure, it has low system efficiency, high energy consumption and poor balance performance. Production data show that the power consumption of the artificial lift systems is accounts for 30% of the total energy consumption in the oilfield [1]. With the continuous development of oil fields, the capacity for well fluid is gradually decreasing. Thus increasing the number of pumping units or oil wells is an effective way to raise production, which will inevitably require higher energy consumption [2] and cause a troublesome contradiction between oil well exploitation and energy consumption [3].

In order to keep reducing the energy consumption of oil wells, scholars all over the world have made extensive energy-saving research effort on the mechanical structure and the motor, such as low-speed permanent magnet synchronous motor [4], variable speed motor [5] and intelligent control [6] in conventional beam-pumping units. Du et al. [7] developed an automatic adjustment system for beam balance to enhance the balance effect and Zhao et al. [8] proposed a hydraulic balance system. A large number of other late-model pumping units have been invented, providing theoretical and practical support for the sustainable development of oil fields.

On the whole, the reliability and durability of some new artificial lift devices are still worse than conventional pumping units. So most of the researches on the energy-saving technology of pumping units still focus on the mechanical structure or intelligent control of conventional pumping units, such as the variable speed drive and its save mechanism by Song et al. [9], the beam follow-up balance during the working by Yang et al. [10], and a flywheel energy storage by Han et al. [11]. The authors in this paper hold that the main causes of the high energy loss in the conventional pumping units are high inertia of moving parts, the large starting power and the poor balance performance. Taking appropriate steps to reduce the inertia of the moving parts and improve the balance performance is the key to reducing the energy consumption.

In recent years, some scholars studied deeply on the application of hydraulic system driven pumping unit. For instances, a mechanical-hydraulic torque coupling transmission mechanism is adopted to realize the energy storage and release through the hydraulic system and proved a good energy-saving effect [12], the dynamic power compensation method is adopted to realize the self-adaptive adjustment and better speed accuracy of the hydraulic pump [13], particular types of hydraulic oil were selected for the hydraulic pumping unit [14] and etc. Obviously, the application of hydraulic drive in pumping unit does have advantages and thus a good application prospect.

In this article, a new type of hydraulically driven direct-balanced pumping unit is proposed against the shortcomings of the beam pumping unit system, such as low transmission efficiency, complicated stroke adjustment and poor balance effect. The hydraulic cylinder is used to directly drive the beam to swing, which removes the transmission mechanism of belt, reducer, four-bar linkage, etc. The balance weight box is directly mounted on the link end, and balance adjustment is more convenient by adjusting the balance weight body in the balance weight box. At the same time, an angle sensor is installed on the central shaft of the beam of the pumping unit to monitor the swing angle of the beam, which determines the reversal of the hydraulic system, and the hydraulic cylinder displacement and the stroke of the pumping unit is stepless adjustable.

With the above methods, the hydraulically driven beam-pumping unit can be produced or modified from the old one. It has realized improving the efficiency of the pumping unit system, stepless adjustment of the stroke, and convenient balance adjustment; futhermore, it greatly reduces the amount of steel consumption of the pumping unit. So it can shorten the production cycle and reduce the manufacturing cost of the pumping unit. The research results would be conducive to promoting the sustainable development of oil field.

## Dynamic analysis of hydraulic drive pumping unit

### A. Kinetic analysis model

**Fig 1:**
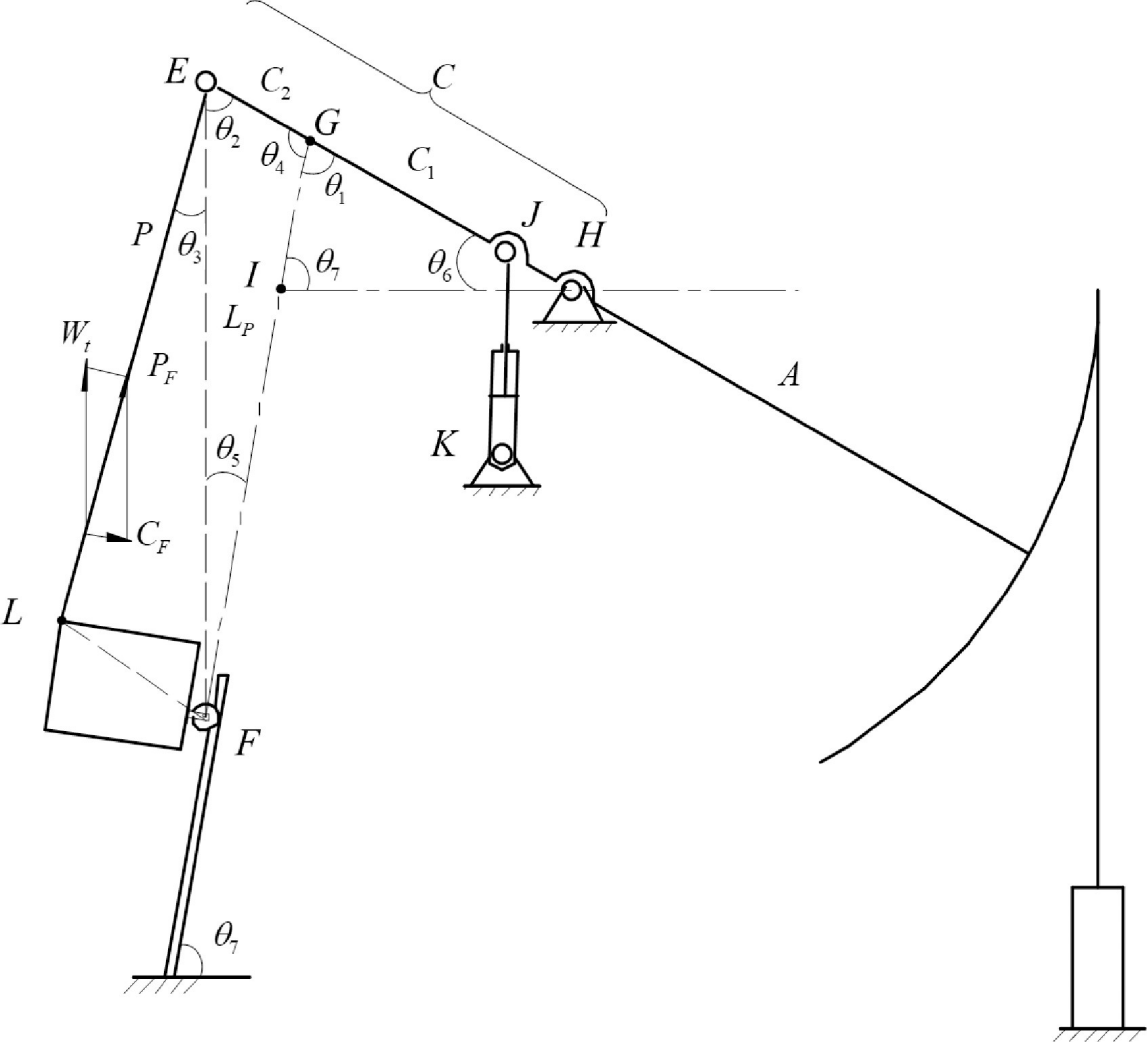
Dynamic analysis model of hydraulically driven beam-pumping unit. In Fig 1, where: *A*—the fore-arm length, mm; *P*—the link length, mm; *W*_*t*_—the balance weight, kN; *C*_*F*_—the guide rail contact force kN; *P*_*F*_—the link force kN; *C*—the rear-arm length, *C* = *C*_1_ + *C*_2_, *C*_1_ is the length of the line *GH* and *C*_2_ is the length of the line *EG*, mm; *L*_*p*_—the length of the line *EF*, mm; *θ*_1_~*θ*_5_—the auxiliary angles for analyzing its balance characteristic, rad; *θ*_6_—the constant angle of beam to horizontal line on the down die point, rad; *θ*_7_—the sliding guide tilt constant angle, rad.

The analytical model of suspension motion, balance weight torque, and contact force on the guide rail of the hydraulically driven beam-pumping unit is shown in Fig 1.

In order to decrease the dynamic load (inertial load and vibration load) of the pumping unit suspension, the reverse calculation method is adopted foe calculation according to the trapezoidal curve law of the suspended velocity. Firstly, the speed curve of the suspension of the pumping unit is established; then the acceleration curve and displacement curve are obtained; at last the movement law of the hydraulic cylinder is determined. Based on the above process, a control strategy for the subsequent hydraulic system can be decided. The control system and method will be studied in the future.

### B. Hydraulic drive pumping unit model

**Fig 2:**
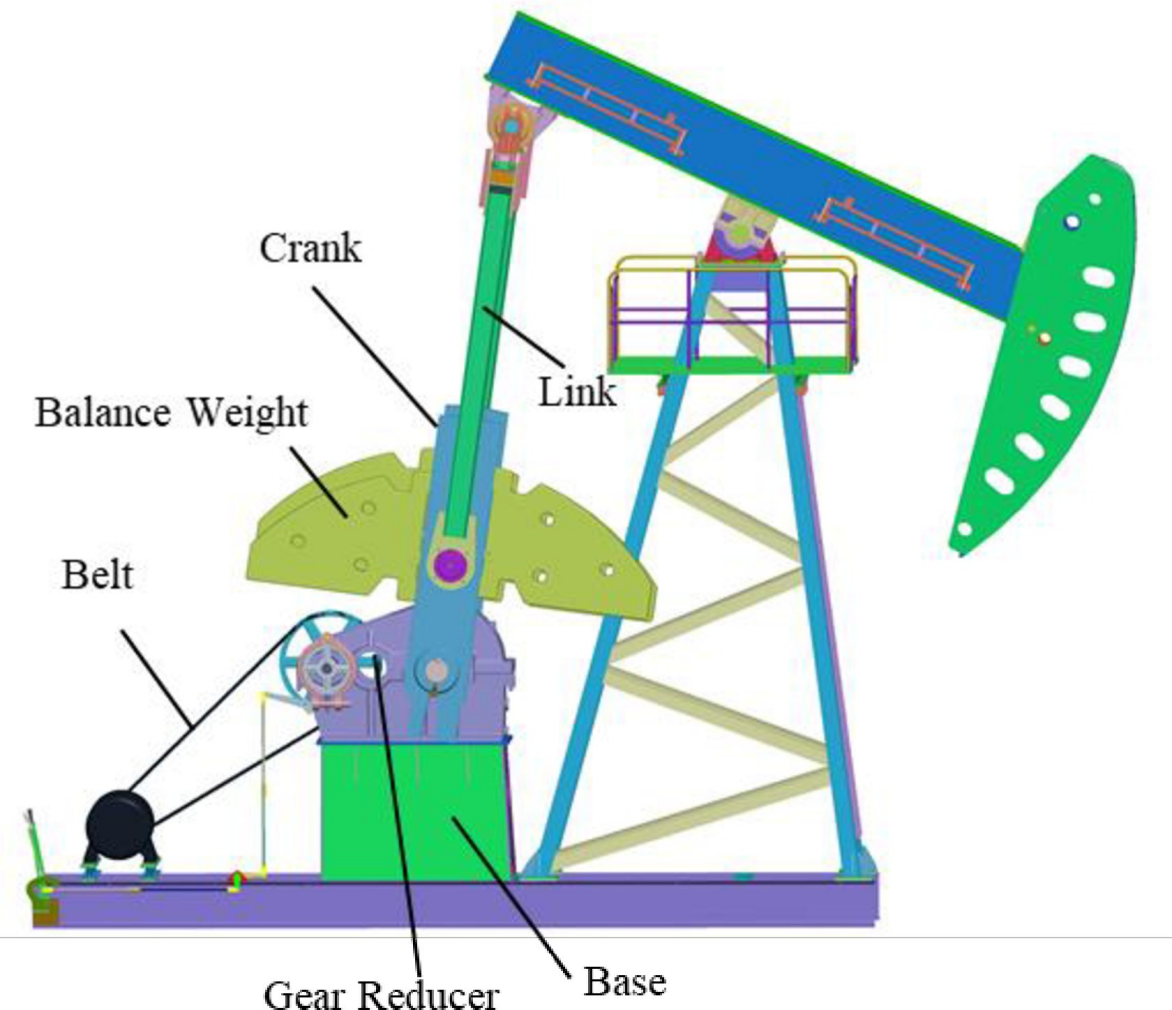
3D Model of conventional pumping unit.

**Fig 3:**
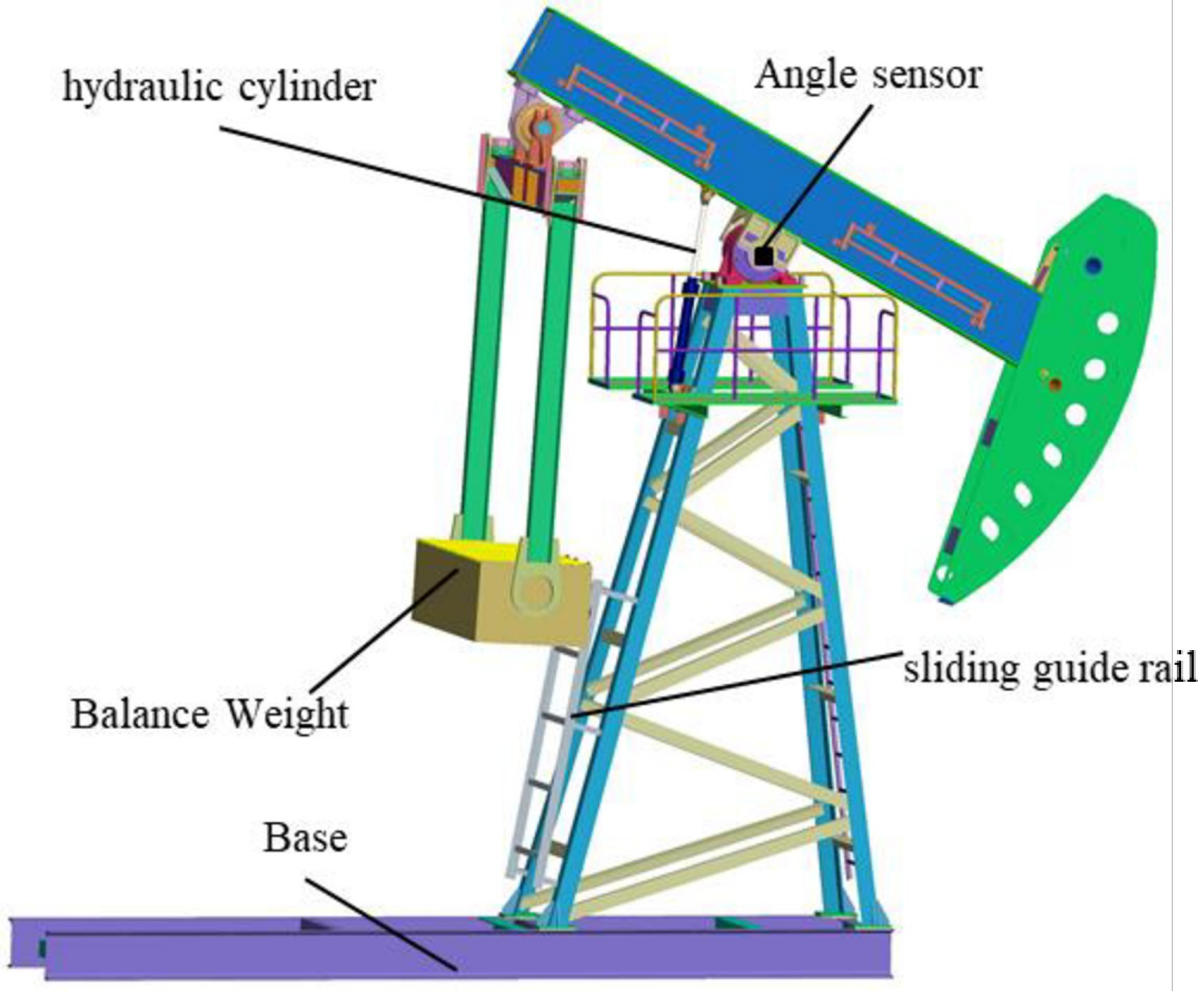
3D hydraulic drive pumping unit.

Compared with the conventional beam pumping unit, the 3d models are shown in Figs 2 and 3. It can be clearly seen from Figs 2 and 3 that the cylinder body, crank, balance weight, belt drive, brake mechanism and other intermediate transmission parts on the pumping unit are removed, and box balance weight, sliding guide rail, hydraulic cylinder and other components are added. Take CYJ10-3-37HB for example, after modification, the hydraulic drive pumping unit can save up to 10 tons of the steel (Crank 2.4t, balance weight 4t, 37H reducer 4t, etc.) or more. Therefore, compared with the conventional pumping unit, the hydraulic drive pumping unit can save nearly 50% of the materials in total.

### C. Hydraulic control principle of hydraulic drive pumping unit

**Fig 4:**
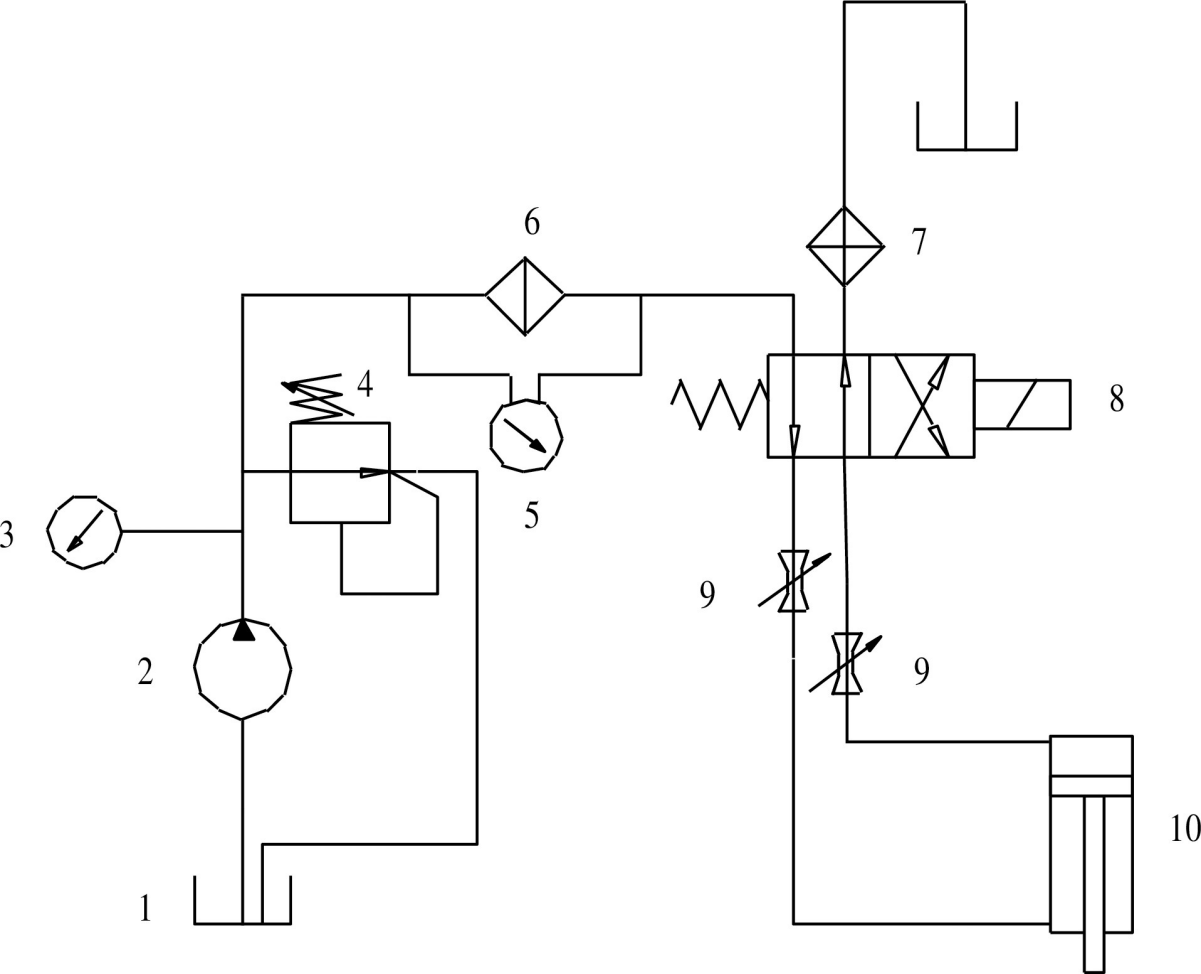
Hydraulic drive schematic. 1-Oil sump; 2-Electric plunger pump; 3-Pressure gauge; 4-Relief valve; 5-Differential pressure signal; 6-High pressure filter; 7-heat sink; 8-two-position four-way solenoid valve; 9-Control valve; 10-Hydraulic cylinder.

The schematic diagram of the hydraulic system of the hydraulically driven pumping unit is shown in Fig 4. The system consists of oil reservoir, electric plunger pump, pressure gauge, overflow valve, differential pressure indicator, high pressure filter, radiator, two-position four-way solenoid valve, regulating valve, hydraulic cylinder, etc. The reversal of the solenoid valve is controlled by the peripheral electrical control circuit, so as to realize the reversing action of the hydraulic cylinder and the up and down movement of the pumping unit.

## Dynamic simulation analysis of pumping unit

The structural dimensions and operating parameters of CYJ10-3-37HB pumping unit, selected as the object, are shown in Table 1.

**Table 1:** Structure size and operating parameters of pumping unit.

**Fore arm length (mm)**	3000
**Back arm length (mm)**	2400
**Link length (mm)**	3350
**Horizontal distance (mm)**	2300
**Crank radius (mm)**	1150
**Center height (mm)**	5290
**Reducer center height (mm)**	1980
**Frequency (min-1)**	3

In order to compare the consistency and comparability of the two simulation results, the calculation processed conducted under the same-well conditions. The well conditions are shown in Table 2.

**Table 2:** Well condition parameters.

**Pump depth (m)**	1000
**Fluid depth (m)**	600
**Rod diameter (mm)**	22
**Tube diameter (mm)**	76
**Pump diameter (mm)**	57
**Watery (%)**	90

**Fig 5:**
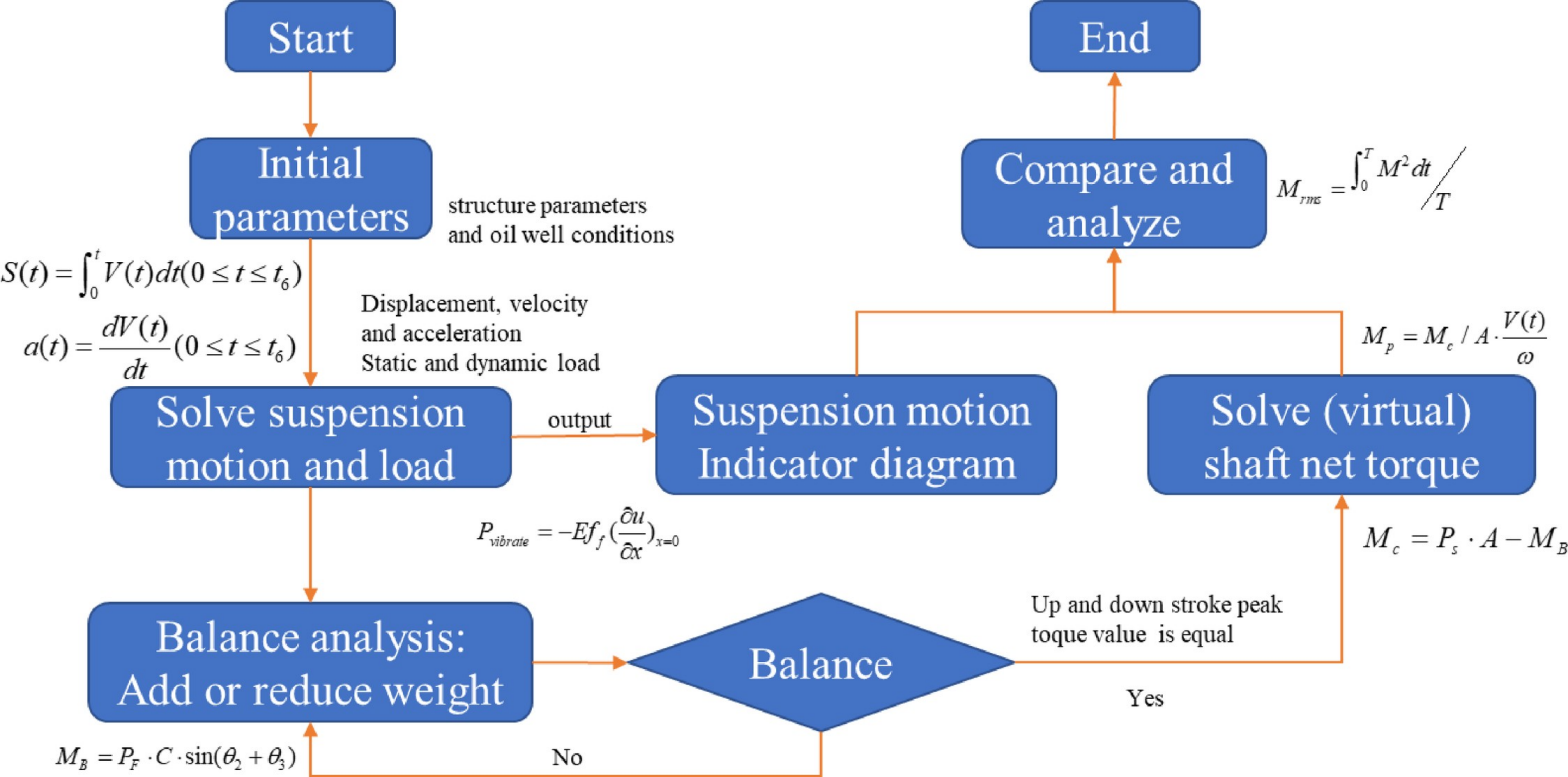
A flow chart for calculation procedures of the solution method.

The flow chart for the calculation procedures of the solution is shown in Fig 5.

### A. Analyses of suspension motion

#### a. Analysis of suspended motion of the hydraulic drive pumping unit

**Fig 6:**
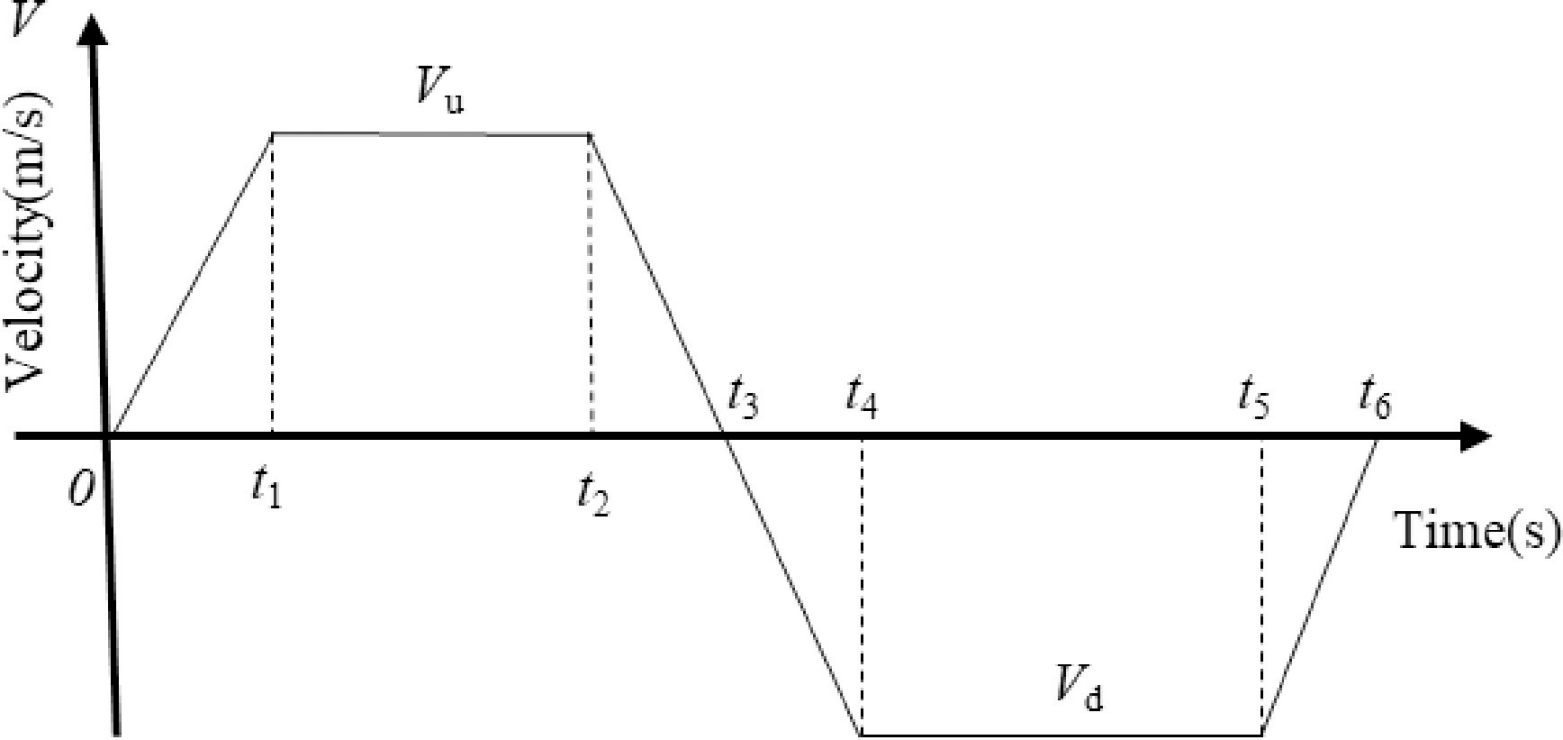
Suspension velocity versus time curve of hydraulically pumping unit.

As is mentioned above, according to the movement law of the suspension of the hydraulically driven pumping unit, the velocity curve is shown in Fig 6. The velocity equation of the suspension is as follows.

$$V(t)=\{\begin{array}{cc} V(t)=V_{u}/t_{1}\cdot t & \left(0\leq t\leq t_{1}\right)\\ V(t)=V_{u} & \left(t_{1}\leq t\leq t_{2}\right)\\ V(t)=V_{u}-V_{u}/\left(t_{3}-t_{2}\right)\cdot t & \left(t_{2}\leq t\leq t_{3}\right)\\ V(t)=V_{d}/\left(t_{4}-t_{3}\right)\cdot t & \left(t_{3}\leq t\leq t_{4}\right)\\ V(t)=V_{d} & \left(t_{4}\leq t\leq t_{5}\right)\\ V(t)=V_{d}-V_{d}/\left(t_{6}-t_{5}\right)\cdot t & \left(t_{5}\leq t\leq t_{6}\right) \end{array}$$ (1)

where:

*t*_1_~*t*_6_—respectively represent the segment running time on the suspension, s; *V*_*u*_、 *V*_*d*_—respectively represent the constant speed of the up and down stroke, mm/s.

Eq (1) can be used to derive the solution equation for the displacement and acceleration of the suspension on the pumping unit, as follows:

$$S(t)=\int_{0}^{t}V(t)dt(0\leq t\leq t_{6})$$ (2)

$$a(t)=\frac{dV(t)}{dt}(0\leq t\leq t_{6})$$ (3)

**Fig 7:**
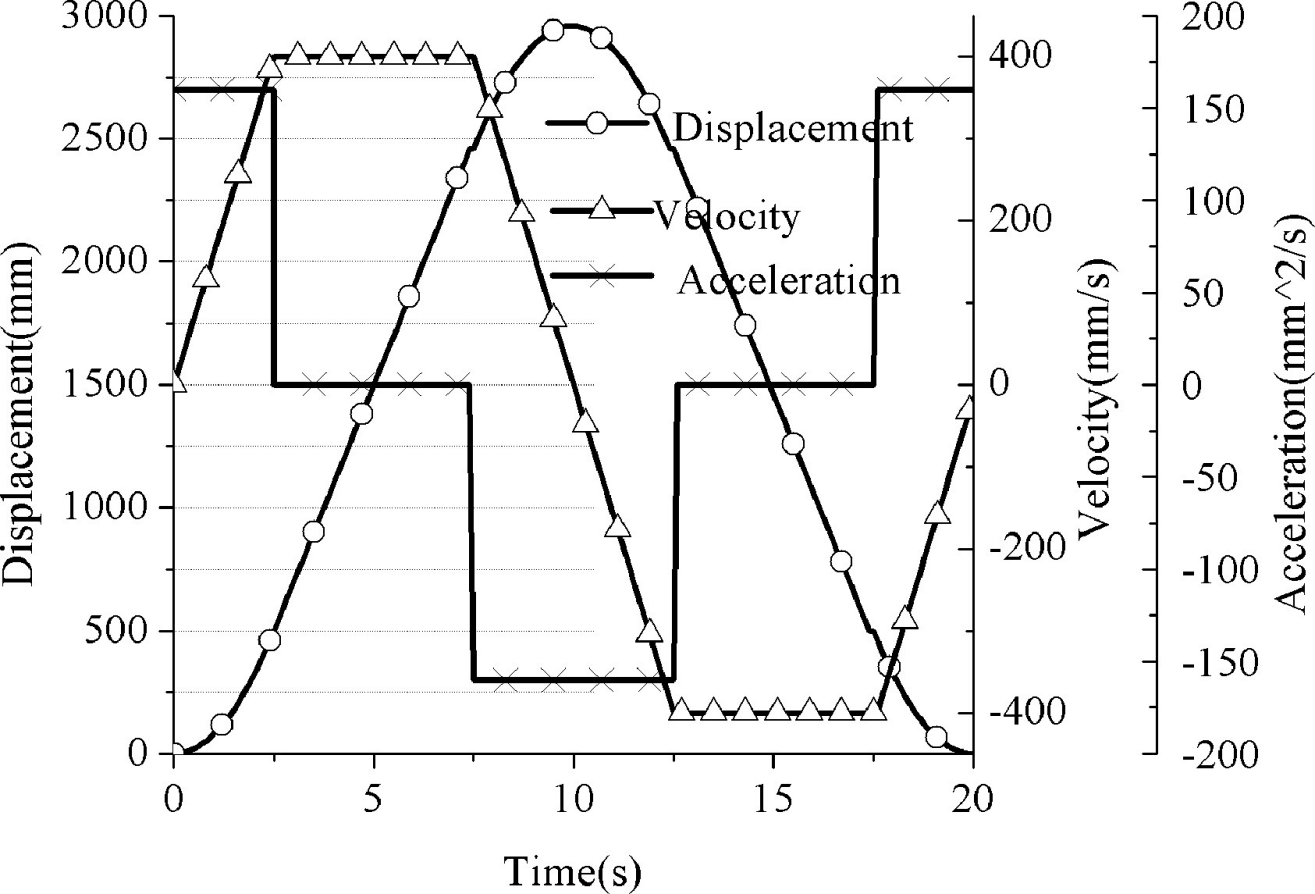
Movement curve on suspension of hydraulic pumping unit.

By solving Eqs (1) to (3), the motion curve on the suspension of the hydraulically driven pumping unit can be obtained as is shown in Fig 7.

#### b. Motion analysis of suspension point of the conventional pumping unit

**Fig 8:**
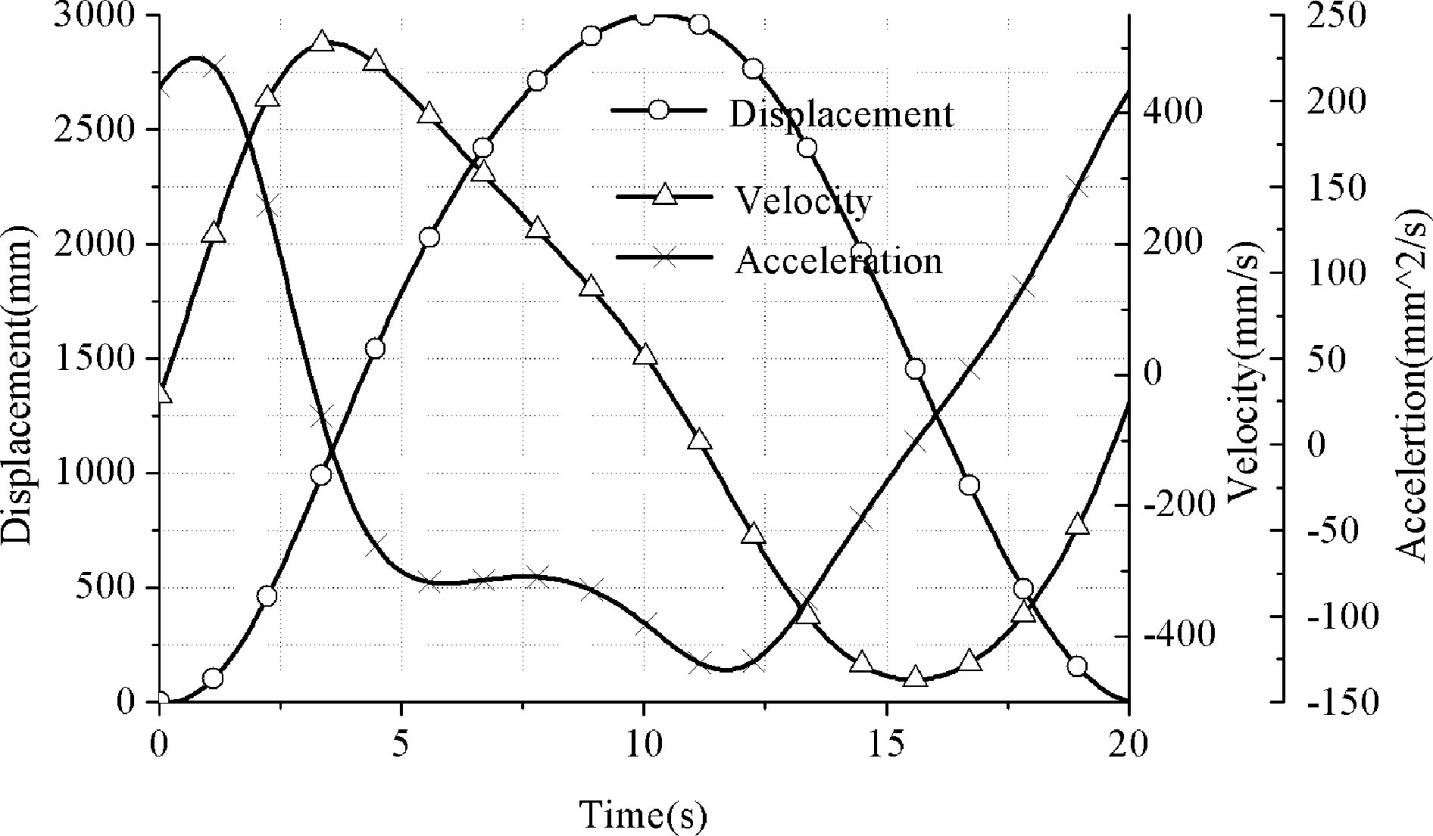
Motion curve of suspension of conventional pumping unit.

In the analysis process of the suspension motion of the conventional beam-pumping unit, the complex vector method is mostly used for simulation analysis and solution. The detailed mathematical model and derivation process can be referred to in the literature [15]. This article does not conduct a detailed analysis of this, only displaying the calculation results as is shown in Fig 8.

By comparing the analysis results of Figs 7 and 8, it can be found that the speed and acceleration of the suspension point of the pumping unit have changed greatly. The maximum speed on the suspension point of the conventional machine is 507.24mm/s, and the maximum speed of the hydraulically driven pumping unit is 400mm/s, which decreases by 21.14%. The maximum acceleration of the conventional one is 224.96mm/s2, and the maximum acceleration of the hydraulically driven pumping unit is 160mm/s2, which drops by 28.88%. The results demonstrate that the hydraulic drive will have a better restraining effect on the dynamic load of the pumping unit suspension point (both inertial load and vibration load). This part of the analysis will be analyzed and explained in Section B.

### B. Comparative analysis of suspension vibration load

For either the static load or dynamic load of the suspension of the pumping units, the calculation methods are the same. The inertial load is related to the acceleration of the suspension, and the vibration load is related to the suspension speed at the end of the elastic deformation of the rod and tube. Assuming the tube end is not anchored, the classical one-dimensional wave equation theory [16] is used in analyzing the vibration load. The longitudinal vibration of the rod string is considered, while the influence of lateral vibration and friction load is ignored.

The one-dimensional wave equation form is as follows:

$$c^{2}\frac{\partial^{2}u}{\partial x^{2}}=\frac{\partial^{2}u}{\partial t^{2}}+v\frac{\partial u}{\partial t}$$ (4)

Where:

*c—*the speed of sound wave transmission in the pole (set c = 5000), m/s;

*x*—the distance from a calculated cross-sectional area in the pole to the suspension, m;

*u*—the displacement of the section relative to the top, m;

*t*—time, s;

*v*—the column damping coefficient (s^-1^)(set v = 1).

Set boundary conditions:

At the start point fixed *u*(0,*t)* = 0.

End not anchored $\frac{\partial u}{\partial x}(L,t)=0$.

In the longitudinal direction of the rod pole, the vibration begins at the end of the elastic deformation, at this time: *t* = 0, so there is:

u(x,0)=0

$$\frac{\partial u}{\partial t}(x,0)=-\psi \frac{x}{L}u_{d}$$

So the solution of the pole vibration is:

$$u=-\psi v_{d}\cdot e^{-\frac{v}{2}t}\times \frac{8}{\pi^{2}}\cdot \sum_{i=1,3,\cdots }^{\infty }\frac{{\left(-1\right)}^{\frac{i-1}{2}}}{i^{2}p_{i}}\sin(p_{i}t)\sin(\frac{i\pi }{2L}x)$$ (5)

Where:

*p*_*i*_—the self-vibration frequency of the system, $p_{i}=\frac{i\pi c}{2L}$;

*v*_*d*_—the suspension speed at the end of the elastic deformation of the up and down stroke, mm/s;

*ψ*—the static deformation distribution coefficient;

*e*—the base of natural logarithm. *L* is the pump depth, m.

So the vibration load on the suspension of the pumping unit can be obtained as

$$P_{vibrate}=-Ef_{f}{\left(\frac{\partial u}{\partial x}\right)}_{x=0}$$ (6)

Where:

*E*—the elastic modulus of steel, MPa;

*f*_*r*_—the cross-sectional area of the rod pole.

**Fig 9:**
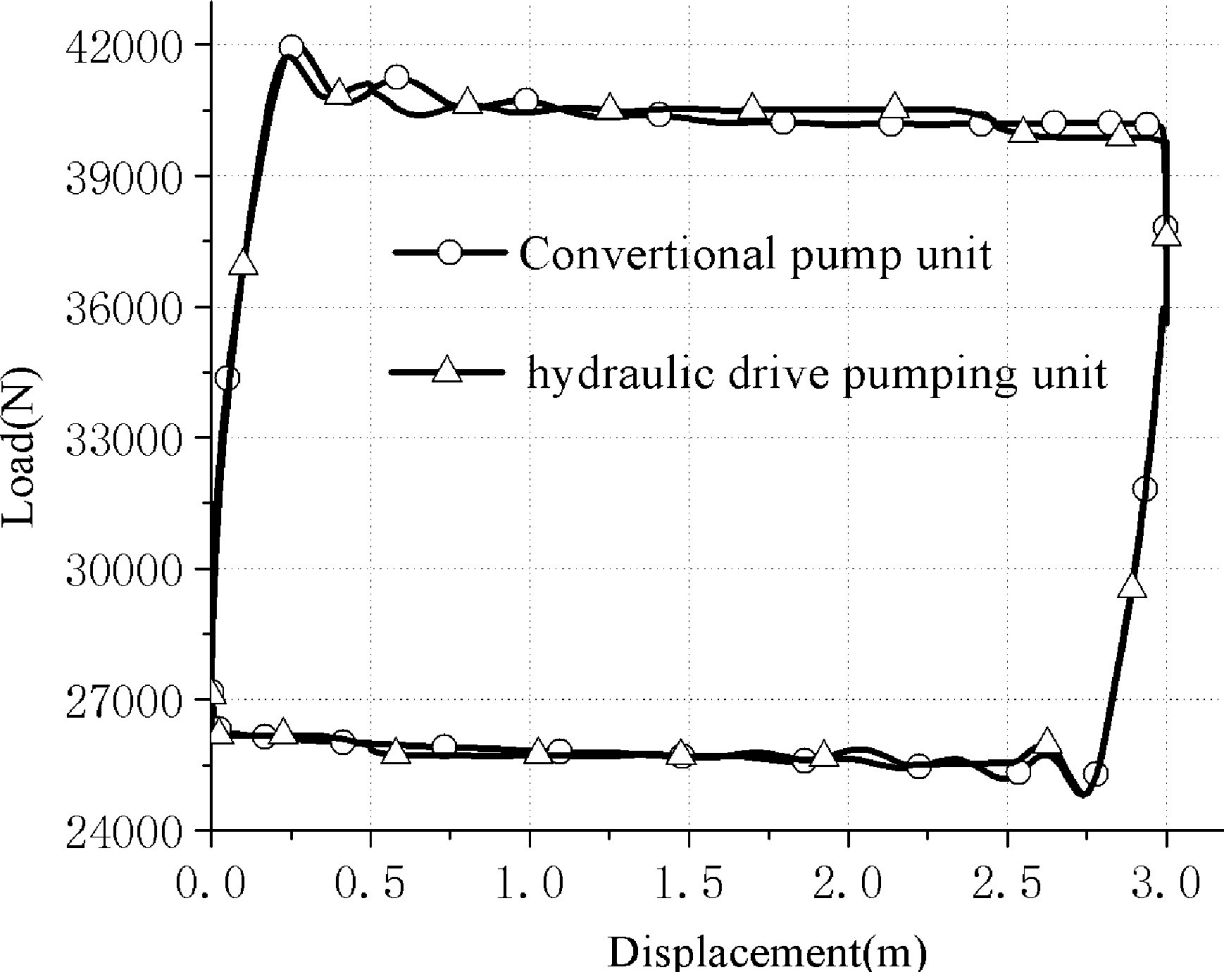
Simulation indicator diagram of conventional and hydraulically driven pumping units.

The simulation calculation results are shown in Fig 9.

By comparing and analyzing the curves of the two power indicator diagrams in Fig 9, it can be obviously seen that the upper stroke load of the hydraulic drive pumping unit has decreased, and the down stroke load has increased. The area of the indicator diagram is reduced, that is, suspension energy consumption is reduced. In fact, the indicator diagram of the hydraulically driven pumping unit is closer to the static indicator diagram, which is conducive to energy saving. The indicator diagrams of the two simulations are similar, mainly because the static load (rod load, oil load, etc.) of the pumping unit suspension is relatively large (upstroke static load 40.52kN, downstroke static load 27.52kN). Under low stroke conditions, the dynamic load of the suspension is relatively small. However, it can still be seen that the hydraulically driven pumping unit still has a better advantage in reducing the dynamic load of the suspension.

### C. Balance characteristic analysis

Qualitative analysis of Figs 2 and 3 can illustrate that the balance weight of a conventional pumping unit is the balance of the suspended load after the conversion of the crank and connecting rod mechanism, which is an indirect balance; The hydraulically driven pumping unit uses the principle of leverage to directly balance the suspension load, which creates a direct balance. Therefore, the hydraulically driven pumping unit has a good balance advantage in the balance mode.

Quantitative analysis: there is no report on the comparison method of the net torque of the conventional beam pumping unit and the hydraulic pumping unit after balance. In this paper, a virtual reducer output shaft on a hydraulic pumping unit is assumed. The speed of the two on the output shaft of the reducer is equal, or in other words, the speed of the motor on the output shaft of the (virtual) reducer is equal. Ignoring the efficiency of the transmission link, a quantitative analysis of the balance characteristics of the two can be achieved by comparing the net torque of the two on the shaft.

In order to compare the balance characteristics of the two, the conventional calculation method of the root mean square value of the torque is used. The greater the root mean square value of the net torque, the worse the balance effect and the higher the motor energy consumption.

**Fig 10:**
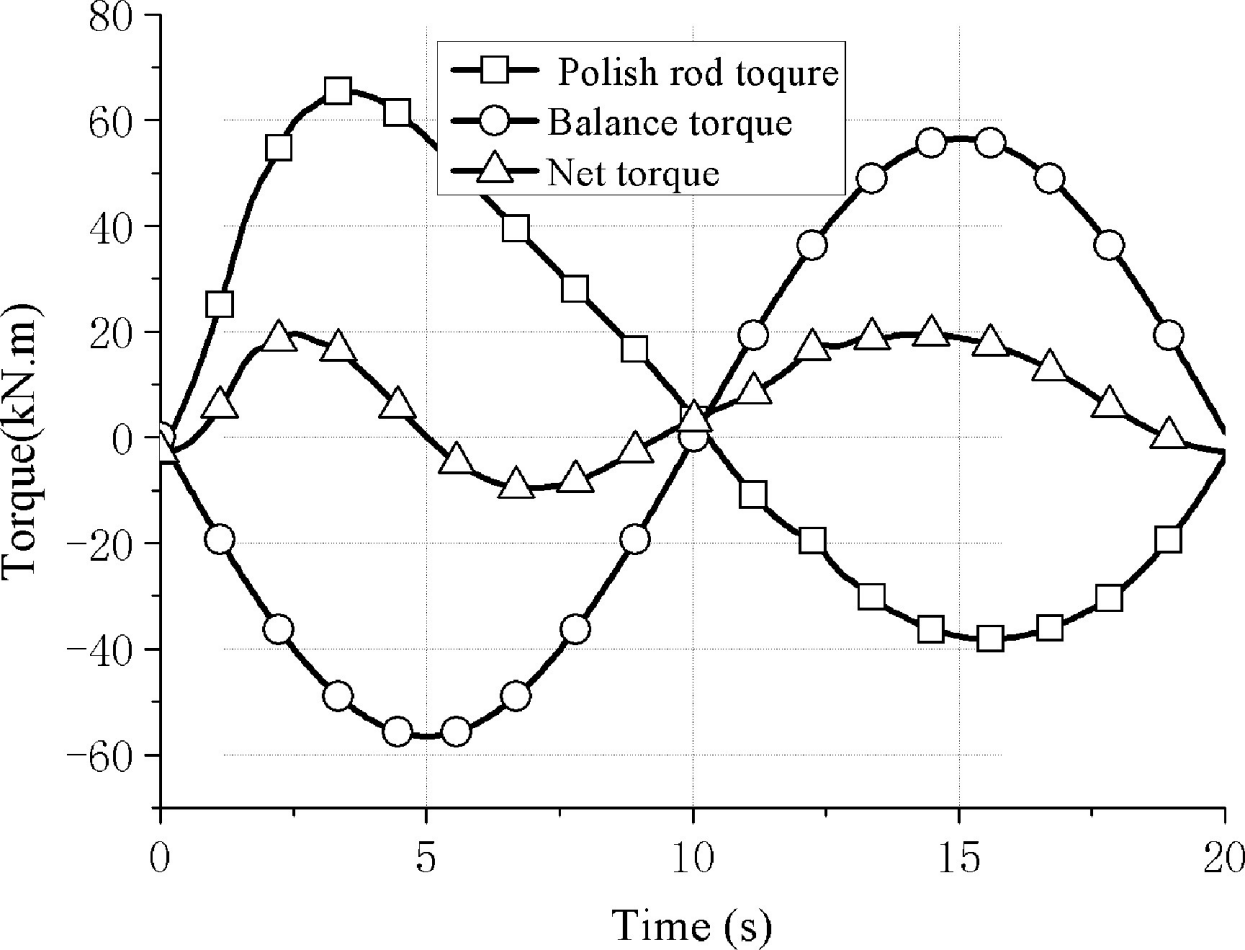
Balance torque curve of conventional pumping unit.

Based on the above pumping unit parameters and well condition parameters for simulation analysis, the balance torque curve on the reducer of the conventional pumping unit in the balanced state is shown in Fig 10.

The torque analysis process of the balance characteristics of the hydraulic pumping unit on the virtual output shaft of the reducer is as follows:

The net torque on the beam center shaft after balance is calculated, in Eq (7).

$$M_{c}=P_{s}\cdot A-M_{B}$$ (7)

Where:

*P*_*s*_—the suspended load, kN;

*M*_*B*_—the torque of the balance weight to the central shaft, kN⋅m.

As to the calculation process of *M*_*B*_, as is shown in Fig 1, the beam angle velocity is

ω=V(t)A (8)

So the angle *θ*_1_ is *θ*_1_ = *π*−*θ*_6_−*θ*_7_+*ω*⋅*t* and *θ*_4_ = *π*−*θ*_1_. Base on sine law, $C_{1}=\frac{l_{IH}}{\sin\theta_{1}}\cdot \sin\theta_{7}$, *C*_2_ = *C*−*C*_1_.

In Δ*ELF*, the length *L*_*p*_ and *θ*_3_ are constant, then in Δ*EFG* we can get $\theta_{5}=a\;\sin(\frac{C_{2}}{L_{p}}\cdot \sin(\theta_{4}))$ and *θ*_2_ = *π*−*θ*_4_−*θ*_5_.

Then *M*_*B*_ is

$$M_{B}=P_{F}\cdot C\cdot \sin(\theta_{2}+\theta_{3})$$ (9)

**Fig 11:**
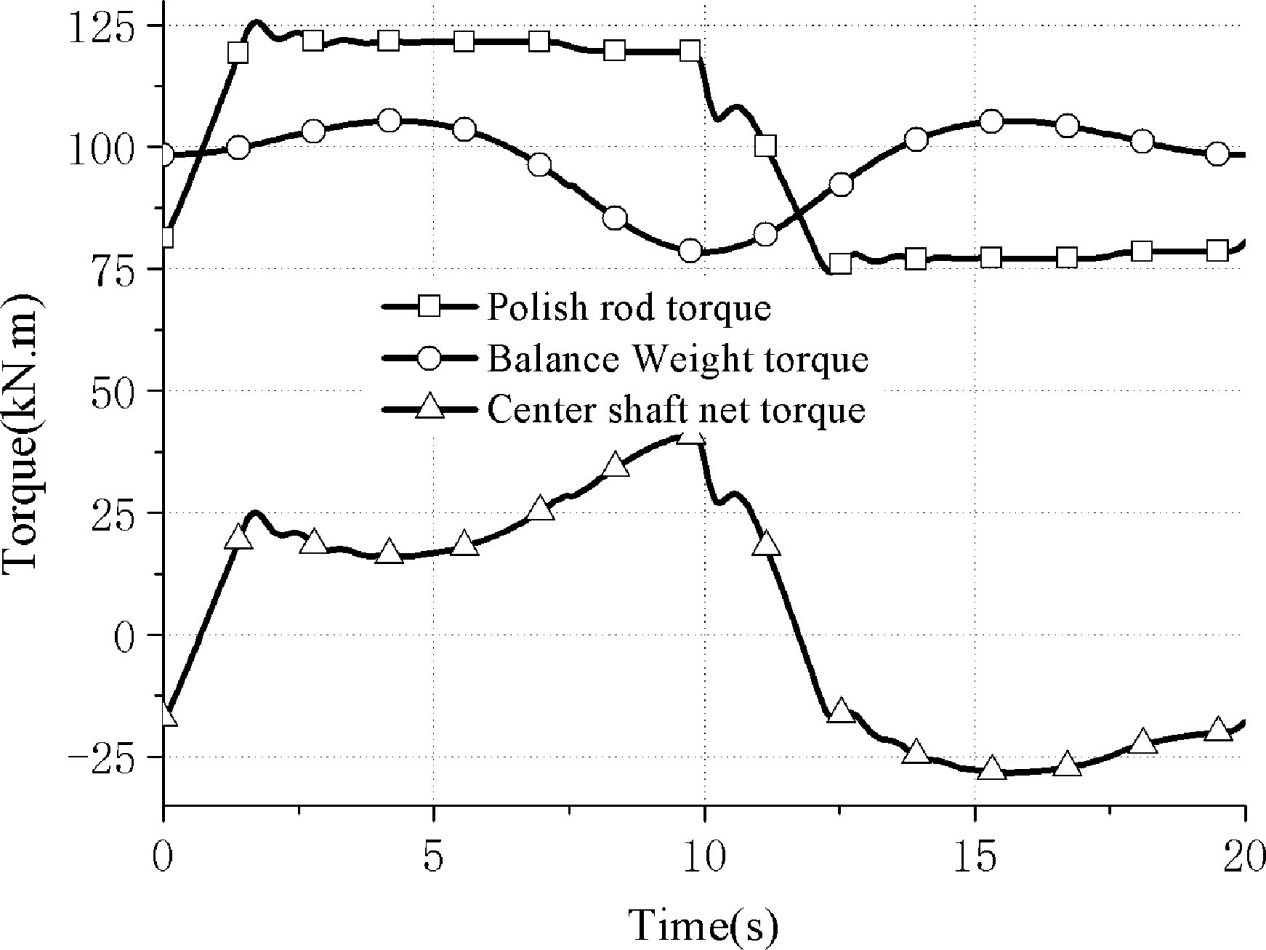
Torque curve of center shaft of hydraulically driven pumping unit.

The theoretical simulation curve obtained is shown in Fig 11.

According to Eq (7) and the previous assumptions, then the torque on the output shaft of the virtual reducer, without the consideration of the system transmission efficiency, is

$$M_{p}=M_{c}/A\cdot \frac{V(t)}{\omega }$$ (10)

Where *V* is the suspension speed, m/s; *ω* is the output shaft speed of the virtual reducer, rad/s.

**Fig 12:**
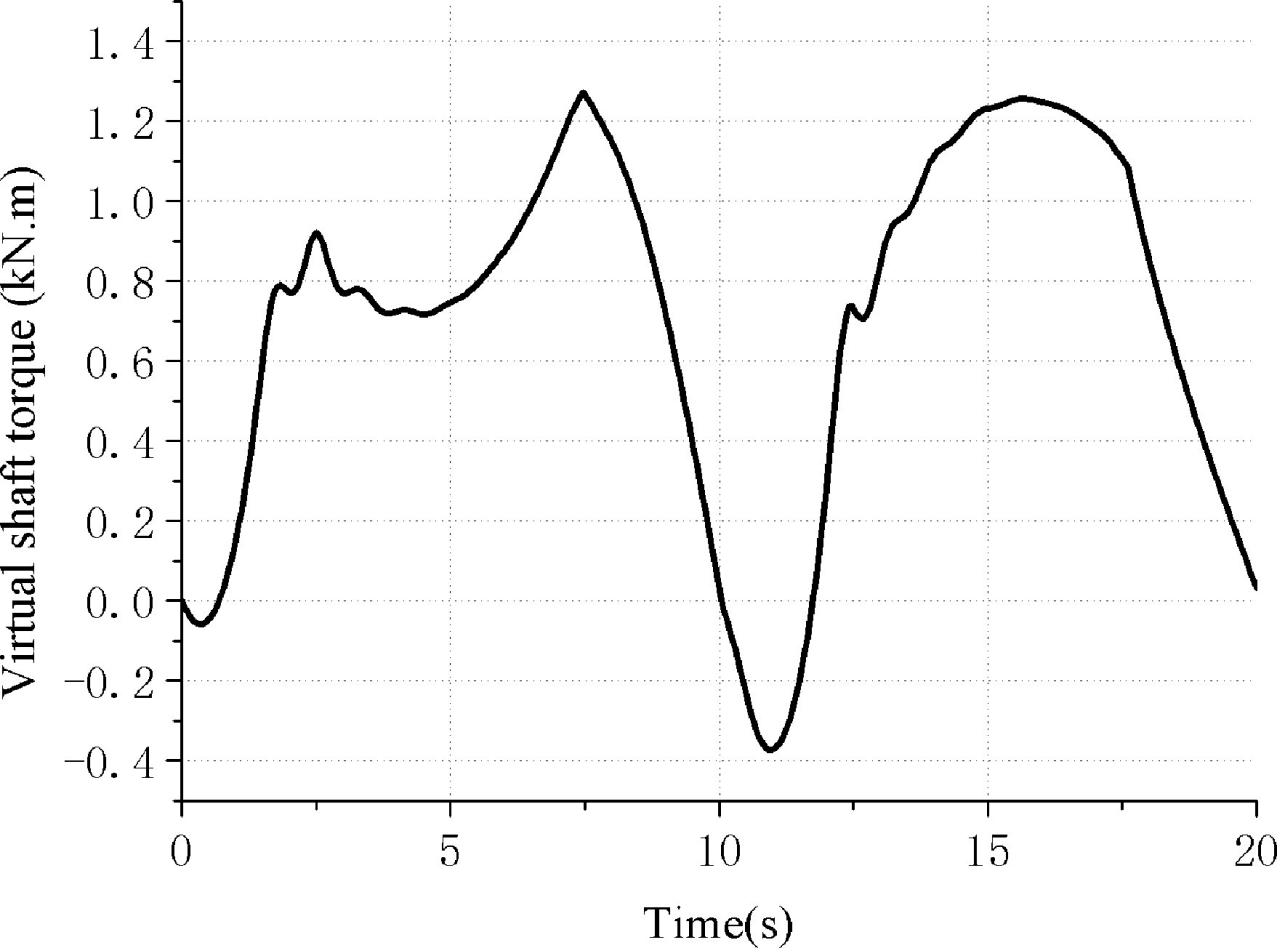
Virtual shaft torque curve of hydraulically driven pumping unit.

The net torque curve obtained on the output shaft of the virtual reducer is shown in Fig 12.

By using the net torque root mean square value to compare the torque curve characteristics in Figs 10 and 12, the root mean square value calculation method is

Mrms=∫0TM2dtT (11)

Where:

*M* is the net torque value, kN⋅m;

*T* is a stroke time, s.

Thus the root mean square value of the net torque curve in Figs 9 and 11 can be obtained, and they are 11.7 kN⋅m and 0.835 kN⋅m respectively. That’s a 92.9 percent drop and the energy-saving advantage is obvious, that the direct balance mechanism has good balance characteristics and energy-saving advantages.

## Results and discussions

**Fig 13:**
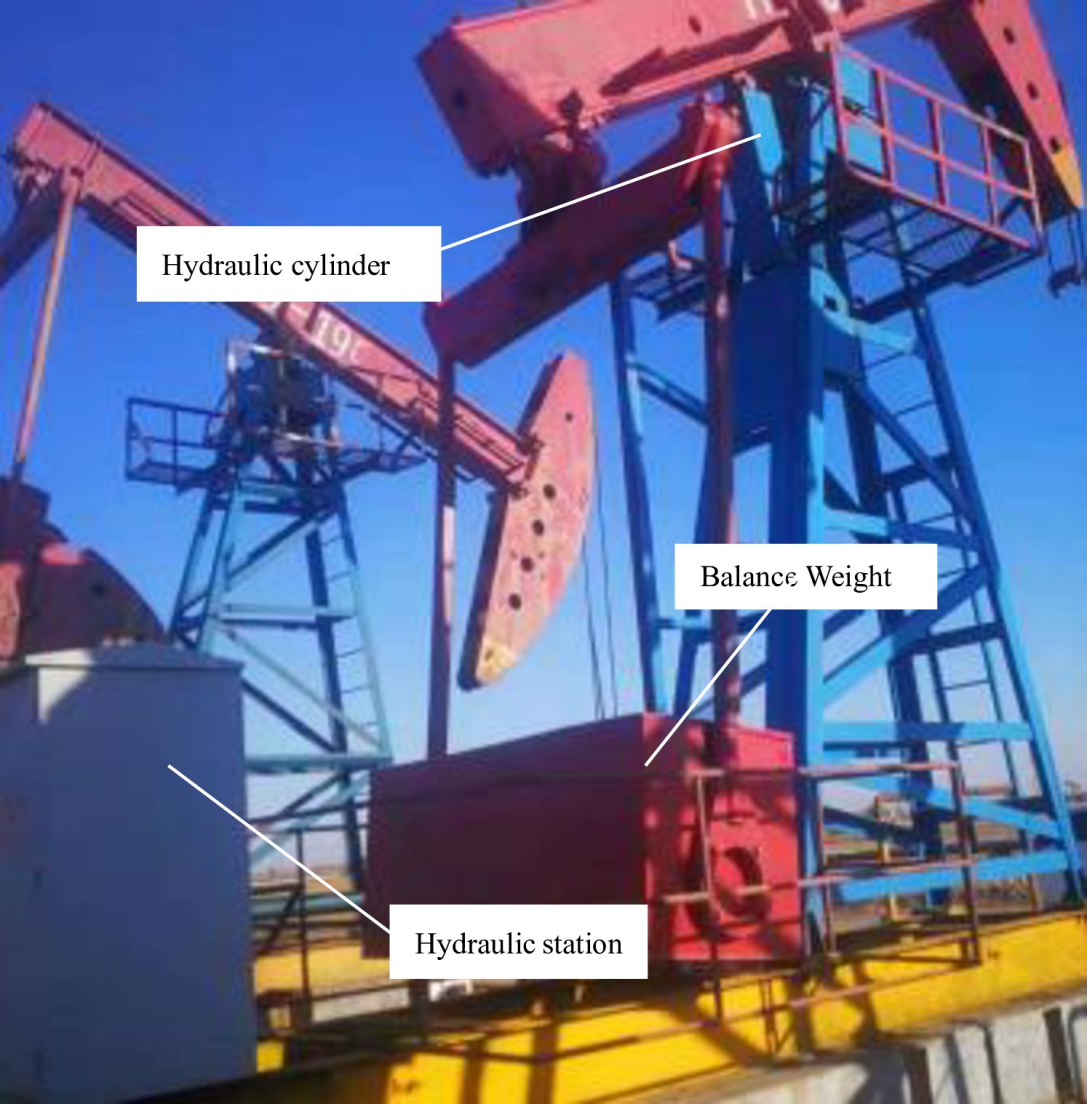
Hydraulic drive pumping unit.

The theoretical research results show that the pumping unit adopting the hydraulic drive direct balance method has good advantages in reducing the suspended load and the net torque of the motor. In order to further verify the results of theoretical research, the designed principle prototype was tested in an oil field in September 2020 as is shown in Fig 13.

The basic data of the well condition are shown in Table 3.

**Table 3:** Oil well condition parameters.

	Conventional Pumping Unit	Hydraulic Pumping Unit
**Pump diameter (mm)**	38	38
**Pump depth (m)**	861.8	860.5
**Submergence (m)**	150.9	149.5
**Rod diameter (mm)**	22	22
**Stroke (m)**	3	3
**Frequency (min^-1^)**	4.0	2.5
**Watery (%)**	84.3	84.7

The test results of the conventional and hydraulic drive pumping unit are shown in Table 4.

**Table 4:** Test comparison results.

	Conventional Pumping Unit	Hydraulic Pumping Unit	Change range (%)
**Production (t/d)**	7.34	7.2	-1.9
**Pump efficiency (%)**	37.45	36.73	-1.9
**P_max_ (kN)**	33.1	31.2	-5.7
**P_min_ (kN)**	20.6	21.5	4.4
**Active power (kW)**	3.43	2.37	-30.9

The test results in Table 4 show that the energy-saving advantages of hydraulically driven pumping units are obvious and the active power save-rate reached 30.9% though slightly reduced liquid production. More importantly, the upstroke load is reduced and downstroke load is increased. To some extent, the experimental results are proved to be consistent with the theoretical analysis. Therefore, the hydraulically driven pumping unit has a beneficial prospect in reducing the area of the indicator diagram and energy-saving.

## Conclusions

This paper presents a new type of hydraulically driven direct balance pumping unit. The mathematical analysis model of the suspension dynamics of the pumping unit is established. The comparison of the movement and the indicator diagram of the suspension between the conventional pumping unit and the new one with hydraulic drive are made. Qualitative analysis and quantitative analysis of the balance characteristics are studied. Through engineering tests, the energy-saving effect of the hydraulic drive pumping unit is verified. Conclusions are thus drawn as below:

1. The maximum speed and acceleration of the suspension of hydraulic pumping unit are reduced by 21.14% and 28.88% respectively.
2. The upper stroke load of the hydraulically driven pumping unit is reduced, and the lower stroke load is increased. This causes the area of the indicator diagram to be reduced. As a result, the indicator diagram of the hydraulically driven pumping unit is closer to the static indicator diagram, which is conducive to energy saving.
3. A method for quantitative analysis of the balance characteristics of hydraulically driven pumping units is given. The quantitative analysis shows that the root mean square value of the net torque of the hydraulically driven pumping unit is decreased by 92.9%. So the energy saving advantage of the new hydraulic pumping unit is obvious.
4. The experimental results show that the hydraulically driven pumping unit has an active power saving rate of up to 30.9%.

## Supporting information

S1 Data (OPJ)
